# Seasonal Variations in Multiple Sclerosis Relapses in Oman: A Single Tertiary Centre Experience

**DOI:** 10.3390/ijerph21101371

**Published:** 2024-10-17

**Authors:** Rashid Al-Shibli, Abdullah Al-Asmi, M. Mazharul Islam, Fatema Al Sabahi, Amira Al-Aamri, Mehwish Butt, Meetham Al-Lawati, Lubna Al-Hashmi, Jihad Al-Yahmadi

**Affiliations:** 1 College of Medicine & Health Sciences, Sultan Qaboos University, P.O. Box 17, Muscat 123, Oman; s129044@student.squ.edu.om (R.A.-S.); allawati01@gmail.com (M.A.-L.); s129223@student.squ.edu.om (L.A.-H.); alyahmadi.jm@gmail.com (J.A.-Y.); 2 Neurology Unit, Department of Medicine, College of Medicine & Health Sciences, Sultan Qaboos University, P.O. Box 17, Muscat 123, Oman; 3 Department of Statistics, College of Science, Sultan Qaboos University, P.O. Box 36, Al-Khoud, Muscat 123, Oman; mislam@squ.edu.om; 4 Department of Operation Management & Business Statistics, College of Economics and Political Science, Sultan Qaboos University, P.O. Box 20, Muscat 123, Oman; a.alaamri4@squ.edu.om; 5 Neurology Unit, Department of Medicine, Sultan Qaboos University Hospital, University Medical City, P.O. Box 35, Al-Khoud, Muscat 123, Oman; m.butt@squ.edu.om

**Keywords:** multiple sclerosis, seasonal variation, multiple sclerosis relapses, climate change, Oman

## Abstract

(1) Background and Aims: The seasonal factors influencing multiple sclerosis (MS) relapses remain elusive. This study aims to investigate the seasonal variation of MS relapses in Oman and compare it globally. (2) Subject and Methods: This retrospective study was conducted on N = 183 Omani MS patients treated at Sultan Qaboos University Hospital, a tertiary hospital in Muscat, Oman, over sixteen-year period (2007–2022). Demographic and clinical data of all MS patients were juxtaposed with the monthly weather data during this period, using descriptive and inferential statistical techniques. (3) Results: Among the N = 183 MS patients studied, 508 relapses were recorded during the study period. The average number of relapses per patient was 2.8 (range: 1–15). There were significant seasonal variations in MS relapse rate, with the highest prevalence in the winter months of January and February. However, no correlation was found between MS relapses and other climatic parameters (humidity, temperature, and rainfall). (4) Conclusion: The seasonal patterns of MS relapses in Oman differ from other parts of the world, which the local clinicians should take into account while diagnosing and making management decisions. The potential impact of climate change on the anomalous changes in the seasonality of MS relapses warrants further investigation.

## 1. Introduction

Multiple sclerosis (MS) is an inflammatory autoimmune disease that affects the central nervous system (CNS). Although the precise pathogenesis remains uncertain, it is believed to result from a combination of genetic, environmental, and immunological factors [1,2].

As per The Atlas of MS, the number of MS patients worldwide has increased from 2.3 million in 2013 to 2.8 million in 2023, reflecting a global MS prevalence of 1/3000 [3]. The prevalence of MS is generally higher in regions farther from the equator [4]. Thus, South and Southeast Asia, Africa, and the Arabian Gulf region (including Oman) have reported lower MS prevalence rates. Oman has an estimated MS prevalence of 15.9 per 100,000 population and adds new cases at a yearly rate of 0.5–1/100,000 [5].

The symptoms of MS have been suggested to have a seasonal influence as in other systemic, neurological, and immune-mediated diseases, such as type 1 diabetes mellitus, rheumatoid arthritis, narcolepsy, and autoimmune encephalitis [6,7,8,9,10]. Among the environmental variables that could trigger or modulate autoimmune symptoms are temperature, humidity, sunlight exposure, vitamin D production, viral infections, and exposure to seasonal allergens and pollutants.

Epidemiological studies have revealed a strong influence of seasonality on MS symptoms around the world and in both the Northern and Southern Hemispheres. However, reports from some countries have differed from the general seasonal trend, e.g., Japan, Portugal, and Ireland [1,11,12,13]. Another contrarian example is Saudi Arabia, where a study based on 219 relapses in 109 MS patients reported the highest prevalence rates in winter months, peaking in January [14].

Apart from the Saudi Arabian study [14], data on this subject remain scarce across the Gulf Cooperation Council (GCC) countries, including Oman. Located in the Arabian Peninsula, these six countries share unique geo-climatic features, including arid deserts, high temperatures, and commonalities in ethnicity and culture. Recent economic growth and acculturation have led to significant lifestyle changes, contributing to emerging health and environmental challenges. The prevalence of MS in the region is on a rising trend, highlighting the urgent need for comprehensive data collection to support informed decision-making and sustainable development.

This study aims to narrow the research gap by exploring the seasonal variation of relapses among Omani MS patients.

## 2. Material and Methods

The data for this study were obtained from a retrospective cross-sectional study in which records of the patients and follow-up data were drawn from the hospital’s electronic medical records, exploring the seasonal variations of MS relapses of patients seen at the neurology clinic at Sultan Qaboos University Hospital (SQUH), Muscat, from 2007 to 2022. The main two tertiary referral centers for neurological disorders in Oman are in Muscat, with SQUH being one of them. Therefore, MS patients seen in SQUH come from all over the country [5].

The MS patients in this study were diagnosed according to the McDonald Criteria [15,16]. Patients without complete follow-up notes (*n* = 11), and those with relapses due to other CNS demyelinating disorders (*n* = 24) were excluded from the study. We also excluded children under 13 years as they were managed in the pediatric department as per the institutional norms, and their details were unavailable for review.

The following information was collected from the hospital’s electronic medical records: (1) demographic data: age, and sex; (2) history related to MS: age at onset of symptoms, age at diagnosis, MS type, current disease-modifying therapies (DMT) and number received, last score on Expanded Disability Status Scale (EDSS); and (3) history of MS relapses: type of the initial relapse and date of each relapse. A relapse was defined as acute or subacute deteriorations in neurological function in the absence of fever or infection, with partial or full recovery [17].

The Research and Development Department of the Oman Directorate General of Meteorology supplied the long-term monthly data on humidity, temperature, rainfall, and ultraviolet radiation (UVR). These data, collected from 11 weather stations across the country, represent the mean monthly values for each weather parameter, recorded from January 2007 to December 2022.

Ethical approval (MREC # 2828 dated 13 July 2022) was obtained from the Medical Research and Ethics Committee of the College of Medicine & Health Sciences, Sultan Qaboos University. The research was carried out in accordance with the Code of Ethics of the World Medical Association (Declaration of Helsinki).

### Statistical Analysis

The data were analyzed using IBM SPSS Statistics for Windows, version 27 (IBM Corp., Armonk, NY, USA) and presented as mean values with percentages and ranges. We analyzed the number of relapses that occurred during the 16-year period under study. The study considered the number of MS relapses as the outcome (or response) variable, while the patients’ demographic and clinical characteristics, months and seasons of occurrence of relapses, and weather conditions were regarded as predictors. The seasons were classified as follows: spring (March–May), summer (June–August), autumn (September–November), and winter (December–February). Both descriptive statistics (frequency, proportion, mean, and standard deviation) and inferential statistics (analysis of variance (ANOVA), correlation, and regression) were employed for data analysis. Frequency distribution was used to describe the characteristics of the patients across a set of demographic and clinical characteristics. ANOVA and F-test were used to evaluate the univariate association of seasonality with quantitative variables, such as the number of relapses and the patients’ characteristics. To identify the predictive validity of seasonality and weather conditions on the number of relapses—after controlling the confounding effects of patients’ background characteristics—a multiple regression technique using the generalized linear model (GLM) approach was employed. Since our response variable (i.e., number of relapses) was a highly positively skewed (variance > mean) count variable, it was necessary to apply an appropriate count regression model for data analysis instead of an ordinary simple linear regression model. For analyzing such over-dispersed count variables (variance > mean), the negative binomial (NB) regression model is considered more suitable than the Poisson regression model [18]. In NB regression, we used the incidence risk ratio (IRR) with a 95% confidence interval (95% CI) to find significant predictors of relapses.

Before fitting the regression model, we checked for potential multicollinearity (linear dependence among independent variables) which could significantly affect regression coefficient estimates by reducing their precision and weakening statistical power. To assess multicollinearity, we used both the correlation matrix and variance inflation factor (VIF) method. Our analysis revealed that all the correlation coefficients were small (less than ±0.5) and VIF values were less than 5, confirming the lack of significant multicollinearity. A *p*-value < 0.05 was considered significant.

## 3. Results

### 3.1. Patient Characteristics

The participants selected for the study comprised N = 183 individuals, of whom 125 (68.3%) were female. The majority (62%) were below 40 years of age. The average age of the cohort was 37.7 ± 9.1 years. The mean age when MS was diagnosed was 29.1 ± 8.0 years [Table 1]. The average duration of the disease was 10.8 years. Most patients (84%) had relapsing-remitting type MS. Three-quarters (75.4%) of the patients received DMTs, mainly orally (42%), followed by injections (21%). The initial relapse of 34% of the patients was supratentorial in nature, 30% had optic pathway dysfunction, 26% had brainstem-cerebellum involvement, and 23% had spinal cord involvement [Table 1].

### 3.2. Relapses and the Associated Predictors

**Figure 1:**
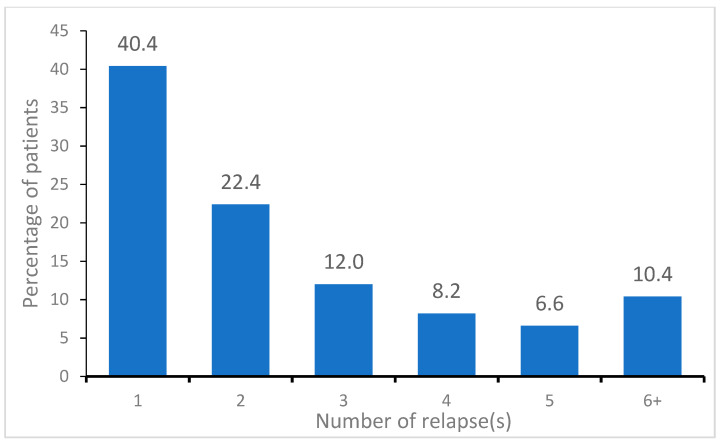
Distribution of multiple sclerosis patients by number of relapses per patient during 2007–2022. (N = 183).

During the 16-year study period (January 2007 to December 2022), there were 508 incidents of relapse in 183 patients, representing 2.8 relapses per patient. All the patients had at least one relapse. About 40% of patients had only one relapse, 22% had two relapses, and the remaining 38% had three or more relapses [Figure 1].

Table 2 presents the results of ANOVA and the NB regression analysis of the count of relapses across a set of background characteristics of patients. The number of relapses significantly increased with the duration of the disease. Patients suffering from MS for more than 10 years had a 1.6 times higher risk of relapses than those suffering from MS for less than 10 years (IRR = 1.56, 95% CI: 1.02–2.39, *p* = 0.039). Types of MS were also significant predictors of relapses. Relapse was more likely to occur among the patients with relapsing-remitting than progressive MS (IRR = 1.91, 95% CI: 1.03–3.78, *p* = 0.042). Relapses increased significantly with the number of DMTs used. The likelihood of relapse was 3.7 times higher among the patients who used four or more DMTs compared to patients with no DMT used (IRR = 3.68, 1.27–10.62, *p* = 0.016). Females were 12.0% more likely to have relapses than males, while younger patients (<40 years) had more relapses than older patients, but both these differences lacked significance.

### 3.3. Seasonality in Relapses

**Figure 2:**
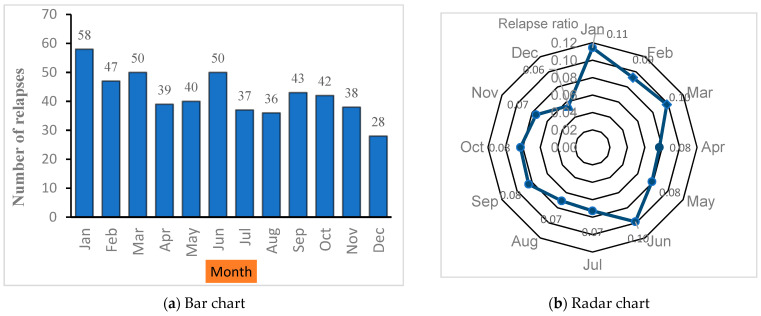
(**a**) Bar chart of monthly variations in total MS relapses among Omani patients, 2007–2022. (**b**) Radar chart showing the proportion of total monthly MS relapses versus expected relapses, 2007–2022.

Table 3 presents the summary of relapses number by month and the corresponding average weather condition variables in Oman during 2007–2022, viz., humidity, temperature, rainfall, and UVR. It is evident from Table 3 and Figure 2 that the highest number of 58 (11%) relapses occurred in January, followed by March and June with 50 (10%) each. The fewest relapses (28, 6%) occurred in December. The monthly average number of relapses in January was 5.0, while in December, it was only 2.3. Overall mean relapses were 3.5 per month in the sixteen-year period studied.

To examine the association between relapses and seasonality (months and seasons), we fitted separate NB regression models with months and seasons as separate explanatory variables, after controlling the confounding effects of the background characteristics of the patients. The results of the NB regression analysis are presented in Table 2 and Table 4. These results indicate a significant effect of specific months as well as seasons on the number of relapses after controlling the background characteristics of the patients. For example, the relative risk of occurrence of MS relapses in January was much higher than in December (IRR = 4.27, 95% CI: 1.59–11.41, *p* = 0.004) [Table 4]. On the other hand, the winter season overall showed a significantly higher risk of relapses compared to Autumn (IRR = 2.08, 95% CI: 1.23–3.34, *p* = 0.006) [Table 2].

### 3.4. Climatic Factors and MS Relapses

**Figure 3:**
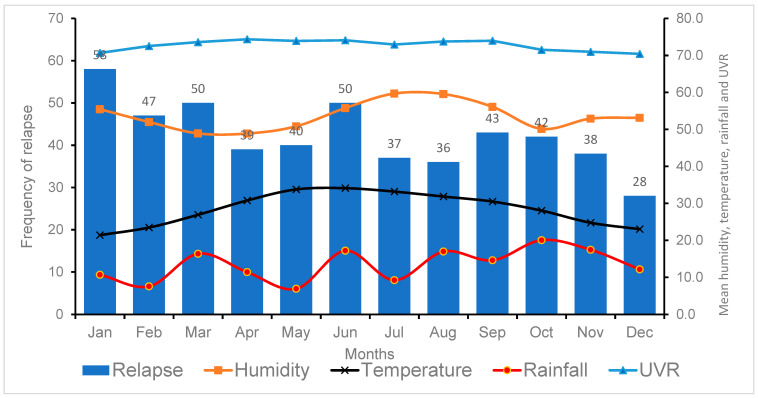
Bar chart showing the frequency of multiple sclerosis relapses per month combined with three-line graphs showing monthly average humidity, temperature, and rainfall in Oman during 2007–2022.

The monthly variations in the average weather conditions in Oman are evident from Table 3 and Figure 3. The most prominent variable was temperature, with a mean annual variation of 45.8%, ranging from a low of 21.4 °C in January to a high of 34.1 °C in June. UVR, the least changing variable, remained very high throughout the year. The mean humidity was wavelike, with two annual highs (winter and summer) and two lows (spring and autumn), but always stayed within the ‘human comfort zone’ of 40–60%. The mean rainfall remained low throughout the year, albeit with a mild sinusoidal pattern. We could not identify any consistent relationship between any of these weather variables and the number of relapses, as evident from Figure 3.

Correlation analysis between relapses and weather conditions revealed no significant association between relapses and weather conditions [Table 5]. There is a weak negative correlation between relapse rate and humidity (r = −0.103, *p* = 0.75) and temperature (r = −0.186, *p* = 0.56). There is no correlation between relapse and rainfall (r = 0.006, *p* = 0.98). UVR shows a very weak correlation with the number of relapses (r = 0.079, *p* = 0.80).

## 4. Discussion

This study investigated a possible association between seasonal variations and the number of relapses in a sample of 183 MS patients between 2007 and 2022 in Oman. Several studies have explored the influence of seasonal triggers for autoimmune responses in diseases, such as rheumatoid arthritis and multiple sclerosis [19]. While individual studies from different parts of the world have identified locally valid relationships, a universally valid relationship between seasonal variations and MS relapses remains elusive [1].

### 4.1. Seasonal Variations

The current study found no significant association between MS relapse rates and humidity, temperature, rainfall, or UVR. Several previous studies have also been inconclusive regarding seasonal variations in MS relapses. An Argentinian study with 431 MS patients failed to find any noteworthy association [20]. Similarly, no significant climatic association emerged in a Portuguese retrospective investigation over four years on 414 relapses in 249 RRMS patients [11]. A Japanese study with 172 MS patients revealed a tendency for more relapses in optic spinal and brain lesions to occur in the warmest (July and August) and coldest months (January and February), although this trend was not statistically significant [12].

On the other hand, some studies found significant weather correlations. In a study conducted on 178 MS patients in the American state of Arizona, which has a desert climate, relapse rates were high in April, June, and July, but were the lowest in May (again breaking the pattern for summer months) and in December [7]. A Danish study investigated the annual rate of relapses (ARR) in their cohort of MS patients who had a total of 16,083 relapses between 1996 and 2020 [21]. They found that the annualized relapse rate (ARR) peaked at 0.203 in February and bottomed out at 0.166 in July, suggesting that increased exposure to sunlight and heat during summer may have lowered relapse rates in July [21]. A Scottish study that reviewed 7098 admissions for MS cases from 1997 to 2009 found the highest numbers of admissions were in late spring to midsummer (April–June), while the lowest were in spring (March) and autumn (October) [22]. A recent systematic review and meta-analysis of 24 studies (mostly from the Northern Hemisphere) with a total of 29,106 MS patients found that the overall relapse rates were higher during spring (March–April) and lower during late summer and autumn (August–November) [1]. From the above, it is evident that different studies have also shown different results regarding the seasons or months that had a higher number of MS relapses during the year.

In Oman, we found a larger number of relapses during winter months, particularly January. A study from neighboring Saudi Arabia [14] also found January to be the peak month of relapses in a sample of 106 patients who had 219 relapses. The lowest prevalence of relapses in Saudi Arabia occurred during the summer season. This is somewhat similar to the prevalence pattern in our study which showed a gradual reduction from February to year-end, except for a spike in June [Figure 3]. It is possible that the Arabian Peninsula may have its own unique regional climate triggers for MS relapses [23].

The findings of our study are presented, alongside those of global and regional studies, in Table 6.

A review based on the MSBase Registry [25] involving 9811 patients with 32,762 relapses across 30 countries discovered that in both hemispheres, relapses followed an annual wave-like pattern that peaked in early spring and bottomed out in autumn. Another significant finding was that the greater the distance from the equator, the shorter the time from one seasonal bottom to the next peak (*p* = 0.028). The authors associated this latitude dependency with the falling ultraviolet radiation (UVR) at higher latitudes.

A German study supported the above finding, with more relapses during late summer and early Autumn [24]. However, Germany’s northern neighbor Denmark [21] showed the opposite pattern with minimal relapses in July. The authors’ explanation that Danish people take their annual vacations in summer and spend more time outdoors appears insufficient because taking summer vacations is a pan-European practice and should be applicable to Germans as well.

Another factor associated with distal latitudes is cold stress which can cause sensitivity in some MS patients. However, it is difficult to establish a correlation between extreme weather events and relapse frequency [21,26]. Individual patients may react differently to similar weather conditions. Therefore, it is essential to consider each patient’s history of response to past weather conditions while evaluating their health [26].

Opposite climatic conditions—summer heat, high UVR, rainfall, and humidity—are also found to influence MS relapses [19]. A study in Southern Brazil reviewed the data of 209 MS patients over 20 years (1990–2010) and found relapse rates peaking in the summer months (December–January), which also had the highest annual levels of temperature, UVR, rainfall, and humidity [19]. However, this apparent anomaly could be explained by the possibility that higher outdoor temperatures may exacerbate symptoms in existing MS patients [27].

Air pollution, especially in urban areas with higher temperatures, may influence disease activity in patients with RRMS. A Serbian retrospective study among 101 RRMS patients with 260 relapses found a significant positive correlation between the rate of relapses and air pollution [28].

Our study explored how climate parameters (rainfall, humidity, temperature, and UVR) impacted MS relapses. Notably, the relapse peak in January is consistent with data from neighboring Saudi Arabia [14]. Previous studies have found the reduced UVR exposure in winter to be a trigger for higher prevalence of MS relapses in winters. Vitamin D helps regulate the immune system by inhibiting proinflammatory cytokines and promoting regulatory T-cells, which helps suppress autoimmune reactions [24,29]. This variable was not included in our study. However, Arabian winters tend to be pleasant and cloudless, leading residents to spend more time outdoors. Conversely, in summer, the extreme heat keeps people mostly indoors. European latitudes, unlike the Arabian Peninsula, receive much lower winter solar radiation due to substantial cloud formations, rain, and snow. Therefore, it may be hypothesized that UVR levels in Arabian winters may not have a significant relationship with MS relapses. This needs to be verified by future studies in the region.

### 4.2. Impact of Global Climate Change

A comprehensive long-term review conducted twelve years ago compared seasonal trends for MS relapses over three distinct decades—the 1980s (1981–1990), the 1990s (1991–2000), and the 2000s (2001–2010). The study found that in the 1990s, relapses peaked in summer (July). Intriguingly, this trend underwent a reversal in the 2000s, with peak relapses occurring in the spring (March) [30]. The authors saw increased pollution and viral epidemics as possible causes for this decadal variation. This raises the possibility that it may also have been an early manifestation of the impact of global climate change.

Indeed, newer studies have hypothesized that the seasonal pattern variations in MS relapses with anomalous and unseasonal weather conditions may have a possible association with global warming. A long-term retrospective study in the U.S. reviewed the cases of more than 100,000 adult MS patients over the period 2003–2017 [31]. It was found that MS-related outpatient visits increased during periods of anomalously warm weather. This association was similar for both sexes, higher in older patients, and varied by season, region, and climate zone. The researchers suggested that individuals with MS may be particularly susceptible to relapses during anomalously warm weather, a phenomenon that is expected to increase with global climate change. Sisodiya et al. (2024) [27] also found a substantial impact of climate change on MS relapses. Thus, the relationship between MS relapses in recent years and patterns of anomalous weather conditions deserves further research, especially in our region.

### 4.3. Limitations

Our study’s limitations include potential selection bias due to its retrospective nature, exclusion of patients who lost contact during the study period, and its cross-sectional design, which made it difficult to establish a causal relationship between seasonal variations and MS relapses. Further, the aggregated weather data that we used may not have accurately reflected regional conditions in different parts of Oman. Being a single-centered study, our findings may be less generalizable for the entire country. Lastly, we did not assess vitamin D levels, which could impact MS relapses. A large pan-GCC region study is warranted to confirm our findings.

## 5. Conclusions

The significant seasonal variations in MS relapse rates underscore the importance of environmental factors in disease expression, which should be considered in clinical practice. In our study, the majority of participants with predominant RRMS were female. Younger patients with long disease duration had frequent relapses. The highest number of relapses occurred in January, followed by March and June, whereas the fewest occurred in December, with the winter season overall showing a significantly higher risk of relapse compared to autumn with a weak negative correlation with humidity levels and temperature. In addition, due attention should be given to potential modifiable factors for alteration of the risk of relapses. Further studies are needed to understand how the seasonal variations in environmental factors, including global warming and climate change, influence MS relapses and disease progression in Oman as well as the entire GCC region.

## Figures and Tables

**Table 1:** The characteristics of Omani patients with multiple sclerosis (MS) (N = 183).

Characteristics	Number	Percent
Number of MS patients	183	100.0
Demographic characteristics		
Sex		
Male	58	31.7
Female	125	68.3
Age in years		
<30	32	17.5
30–39	81	44.3
40+	70	38.3
Mean age	37.7 ± 9.1	
Age at diagnosis in years		
<20	17	9.3
20–29	84	45.9
30–39	65	35.5
40+	17	9.3
Overall mean age at diagnosis	29.1 ± 8.0	
Anthropometric characteristics		
Duration of MS in years		
<10	80	43.7
≥10	103	56.3
Mean duration	10.8 ± 6.6	
MS types		
Clinically isolated syndrome (CIS)	11	6.0
Relapsing remitting (RR)	154	84.2
Secondary progressive (SP)	18	9.8
Nature of initial MS relapse		
Supratentorial lesions present	62	33.9
No Supratentorial lesions	121	66.1
Optic pathway dysfunction	56	30.6
No optic pathway dysfunction	127	69.4
Brainstem-cerebellum involved	48	26.2
No brainstem-cerebellum involvement	135	73.8
Spinal cord involved	43	23.5
No spinal cord involvement	140	76.5
Current DMT Usage		
Naive	45	24.6
Oral DMT	77	42.1
DMT infusion	22	12.0
DMT injection	39	21.3
Overall mean no. of DMTs per patient	1.8 ± 1.1	

Note. DMT: disease-modifying therapy.

**Table 2:** Average number of relapses per patient and the incidence rate ratio (IRR) of the number of relapses, stratified by season and background characteristics of multiple sclerosis patients (N =183).

	Mean Number of Relapses	*p*-Value	Negative Binomial Regression of Number of Relapses		
			IRR	95% CI	*p*-Value
Total	2.8 ± 2.6				
Season		<0.001 *			
Winter	3.53 ± 3.3		2.08	1.23–3.34	0.006 *
Autumn (ref.)	1.54 ± 0.8		1.00		
Summer	2.00 ± 1.1		1.20	0.74–1.76	0.084
Spring	3.25 ± 2.8		1.78	0.78–2.98	0.491
Sex		0.269			
Male (ref.)	2.50 ± 2.2		1.00		
Female	2.96 ± 2.8		1.12	0.74–1.68	0.582
Age at diagnosis (years)		0.159			
<20	2.35 ± 2.2		1.42	0.63–3.90	0.328
20–29	3.14 ± 2.8		1.58	0.72–3.00	0.281
30–39	2.82 ± 2.6		1.47	0.76–3.27	0.218
40+ (ref.)	1.65 ± 1.2		1.00		
Duration of MS (years)		<0.001 *			
<10 (ref.)	1.74 ± 1.2		1.00		
10+	3.65 ± 3.1		1.56	1.02–2.39	0.039 *
Types of MS		0.023 *			
Clinically isolated syndrome (CIS)	1.00 ± 0.0)		1.45	0.44 –4.71	0.540
Relapsing remitting (RR)	2.99 ± 2.7		1.91	1.03 –3.78	0.042 *
Secondary progressive (SP) (ref.)	2.38 ± 1.9		1.00		
Nature of initial MS relapse					
Supratentorial		0.113			
Yes	3.24 ± 3.0		1.10	0.62–1.97	0.674
No (ref.)	2.59 ± 2.4		1.00		
Optic pathway		0.448			
Yes	3.03 ± 3.0		1.17	0.65–2.12	0.592
No (ref.)	2.71 ± 2.4		1.00		
Brainstem-cerebellum		0.894			
Yes	2.77 ± 2.5		1.01	0.57–1.75	0.981
No (ref.)	2.83 ± 2.7		1.00		
Spinal cord		0.143			
Yes	2.30 ± 1.8		1.15	0.61–2.16	
No (ref.)	2.97 ± 2.8		1.00		
Number of DMT used		<0.001 *			
None (ref.)	1.17 ± 0.5		1.00		
1	1.90 ± 1.6		1.37	0.58–3.21	0.432
2	2.90 ± 2.3		1.63	0.67–3.95	0.214
3	3.85 ± 2.8		2.26	1.01–5.93	0.034
≥4	6.26 ± 4.5		3.68	1.27–10.62	0.016

Note. * Significant; MS: Multiple sclerosis; DMT: Disease-modifying therapy; ref.: reference category.

**Table 3:** Monthly summary of mean multiple sclerosis relapses compared with weather variables in Oman during 2007–2022.

Month	Number of Relapses	Mean Humidity (%)	Mean Temperature (°C)	Mean Rainfall (mm)	Mean UVR (mW/cm^2^)
Jan	58	55.4	21.4	10.7	70.7
Feb	47	52.0	23.5	7.5	72.5
Mar	50	48.9	26.9	16.4	73.6
Apr	39	48.9	30.8	11.4	74.4
May	40	50.8	33.8	6.9	73.9
Jun	50	55.8	34.1	17.2	74.1
Jul	37	59.7	33.2	9.2	72.9
Aug	36	59.6	31.8	17.0	73.8
Sep	43	56.1	30.5	14.6	74.0
Oct	42	50.1	28.0	20.0	71.5
Nov	38	52.9	24.7	17.4	71.0
Dec	28	53.1	23.0	12.2	70.4

Note. UVR: Ultraviolet radiation. Source: Department of Research and Development, Directorate General of Meteorology, Oman.

**Table 4:** Negative binomial regression models for the number of MS relapses, showing the IRR by month of diagnosis after controlling the effect of age and sex of the patients.

Months	IRR	95% CI of IRR	*p*-Value
January	4.27	1.59–11.41	0.004 *
February	3.92	1.48–10.34	0.006 *
March	3.03	1.10–8.34	0.032 *
April	3.06	1.09–8.50	0.032 *
May	1.49	0.28–8.10	0.638
June	2.33	0.74–7.38	0.149
July	1.79	0.59–5.43	0.304
August	1.46	0.45–4.76	0.521
September	1.51	0.50–4.55	0.461
October	1.41	0.43–4.57	0.566
November	1.07	0.31–3.66	0.910
December (ref.)	1.00	-	-

Note. * Significant; MS: Multiple sclerosis; IRR: Incidence rate ratio; CI: Confidence interval; ref: reference category.

**Table 5:** Correlation between monthly number of multiple sclerosis relapses and the average humidity, temperature, and rainfall in Oman, 2007–2022.

Weather Parameter	Correlation Coefficient	*p*-Value
Humidity	−0.103	0.751
Temperature	−0.186	0.562
Rainfall	0.006	0.983
UVR	0.079	0.807

**Table 6:** Comparison of results of the current study with those of previous studies regarding the influence of seasonal variations on multiple sclerosis relapses.

Variable	This Study (Oman)	Saudi Arabia [14]	Germany [24]	Argentina [20]	Portugal [11]	Brazil [19]	MS-Base Study [25]
Number of patients	183	182	415	431	414	209	9811
Number of relapses	508	219	355	558	249	996	32,762
Relapses per patient	2.7	1.2	0.9	1.3	0.6	4.7	3.3
Significant seasonal variation	No	Yes	Yes	No	No	Yes	Yes
Season with most relapses	Winter	Winter	Summer	-	-	Summer	Early spring (latitude dependent)
Season with least relapses	Autumn					Winter	Autumn (latitude dependent)
Possible environmental triggers	-	Low vit. D	Low vit. D	-	-	High UVR, Hot, humid	Low UVR and vit. D

## Data Availability

The Corresponding Author can provide further data related to this study upon reasonable request.
